# A prospective cross sectional study of detection of *Clostridium difficile* toxin in patients with antibiotic associated diarrhoea

**Published:** 2018-02

**Authors:** Arun Sachu, Kavitha Dinesh, Ismail Siyad, Anil Kumar, Anu Vasudevan, Shamsul Karim

**Affiliations:** 1 Department of Microbiology, Amrita Institute of Medical Sciences and Research Center, Kochi, Kerala, India; 2 Department of Gastroenterology, Amrita Institute of Medical Sciences and Research Center, Kochi, Kerala, India; 3 Department of Biostatistics, Amrita Institute of Medical Sciences and Research Center, Kochi, Kerala, India

**Keywords:** *Clostridium difficile*, Toxin, Antibiotic associated diarrhoea

## Abstract

**Background and Objectives:**

*Clostridium difficile* infections (CDI) include self-limiting antibiotic associated diarrhoea (AAD), antibiotic-associated colitis, and pseudomembranous colitis. The present study aimed at detecting *C. difficile* toxin in stool samples of patients with AAD and analyzing the antibiotic use and presence of other risk factors in these patients.

**Materials and Methods:**

In this study, which was conducted on 660 samples, a 2- step strategy was used. In the first step, glutamate dehydrogenase (GDH) was detected in stool samples by enzyme-linked immunofluorescent assay (ELFA). In the second step, GDH positive samples were tested for *C. difficile* toxin A and B by ELFA. Nucleic acid amplification test (NAAT) was also performed on few samples that were found to be GDH positive and toxin negative or equivocal by ELFA.

**Results:**

Of the 660 samples screened, toxin was detected in 8.8% (58/660) by ELFA and 9.7% (64/660) by NAAT. GDH was detected in 23.8% (157/660) and toxin in 36.9% (58/157) of the GDH positives. Most of the toxin positive patients were on one or more antibiotics prior to developing diarrhoea. The implicated antibiotics were meropenem, amikacin, colistin and cephalosporins. Diabetes, hypertension, use of proton pump inhibitors, previous hospitalization, malignancy and chemotherapy were found to be the risk factors in our study.

**Conclusion:**

Prevalence of GDH was 23.8% (157/660) by ELFA. Toxin prevalence was 9.7% (64/660). Detection rates of *C. difficile* associated diarrhoea (CDAD) increased with inclusion of NAAT testing by ELFA.

## INTRODUCTION

*Clostridium difficile* is an enteric pathogen that has emerged as the leading cause of antibiotic associated diarrhoea in hospital settings and community populations. In India, *C. difficile* was a neglected pathogen till a few decades ago. However, currently, the well- known associations of this organism have found to be self-limiting antibiotic associated diarrhoea (AAD), antibiotic-associated colitis (AAC), and more serious conditions like pseudomembranous colitis (PMC) and toxic megacolon (1).

*C. difficile* colonises the human intestinal tract after the gut flora has been altered by antibiotic therapy. Approximately, 10% to 35% of antibiotic associated diarrhoea are caused by *C. difficile* infection. Almost all antibiotics can be associated with nosocomial diarrhoea. In hospitals, the most important factor contributing to the *C. difficile* infection (CDI) is the increased use of broad spectrum antibiotics and emergence of hypervirulent *C. difficile* strains known as NAP1/BI/027 (2, 3). Other risk factors leading to development of CDI include advancing age of the patients, prolonged hospital stay, immunodeficiency states and use of antineoplastic drugs and proton pump inhibitors (4). *C. difficile* includes both toxigenic and non-toxigenic strains. Disease manifestations are produced by the toxigenic strains. All toxigenic strains contain toxin B with or without toxin A. Hypervirulent strains produce another toxin called as CDT (*C. difficile* transferase) (5). The clinical manifestations of *C. difficile* infection varies and can range from a mild self-limiting watery diarrhoea to a fatal and fulminant colitis, with potential complications of toxic megacolon and bowel perforation.

The gold standard method in diagnosis is a tissue culture cytotoxicity assay, but the turnaround time is very high. Infectious Disease Society of America has suggested a 2- step strategy to detect *C. difficile* GDH (Glutamate dehydrogenase) antigen by enzyme immunoassay along with a more specific method like toxigenic culture (6). The most important aspect of treating CDI is to stop the inciting antimicrobials and provide adequate hydration and electrolyte replacement. Antibiotics like metronidazole and vancomycin are the mainstay of treatment.

The prime objective of the study was to detect *C. difficile* toxin in stool samples of patients with antibiotic associated diarrhoea and to find the association of antibiotic associated diarrhoea with the usage of specific antibiotics. Medical records were analyzed to find the presence of any comorbidities in patients with CDAD (*C. difficile* associated diarrhoea).

## MATERIALS AND METHODS

This prospective cross-sectional study was conducted in Amrita Institute of Medical Sciences and Research Centre, in Kochi, India, for a period of 30 months, from December 2014 to July 2017. Samples were collected from patients who had been on antibiotic therapy and had subsequently developed diarrhoea.

Inclusion criteria in this study were as follows: (1) Patients had to produce at least 3 unformed stools over a 24- hour period; and (2) they should have received antibiotic therapy within 8 weeks of onset of diarrhoea.

Those patients whose diarrhoea was due to other proven causes and those patients who were on tube feeds were excluded.

This study was approved by the Thesis Protocol Review Committee at Amrita Institute of Medical Sciences and Research Centre. Moreover, informed consent was obtained from the patients. Based on the proportion of enzyme GDH (Glutamate dehydrogenase) in stool samples of patients with diarrhoea reported in an earlier study (7) with 95% confidence and 20% allowable error, the minimum sample size was determined to be 660.

Stool samples were processed within 30 minutes of arriving at the laboratory and were stored at 4°C if immediate processing was not possible. A total of 660 stool samples were screened for the presence of *C. difficile* GDH enzyme by ELFA. Screening was done using VIDAS (bioMerieux) *C. difficile* GDH assay, which is an automated test based on ELFA. The cut- off values for *C. difficile* GDH assay were

Positive- > .10 IU/mL (International Units/mL)
Negative- < .10 IU/mL

The samples negative for GDH enzyme underwent no further testing, and those samples positive for GDH enzyme were screened for the presence of *C. difficile* toxin A and B assay by ELFA. Cut- off values for *C. difficile* toxin A and B assay were

Positive- > 0.37 IU/mL
Equivocal- 0.13– 0.37 IU/mL
Negative- < 0.13 IU/mL.

Those samples positive in both tests were reported as toxigenic *C. difficile* and were reported to the treating physician and infection control department immediately. Microscopic examination under wet mount was done for the toxin positive samples. Samples that were positive for GDH enzyme and negative for toxin were reported as non-toxigenic *C. difficile*.

A nucleic acid amplification test (NAAT) was done using Cepheid GeneXpert *C. difficile* assay on random stool samples, which had high GDH enzyme values and had also toxin values either equivocal or close to the cut- off for equivocal. The test uses automated real-time polymerase chain reaction (PCR) to detect toxin gene sequences associated with toxin producing *C. difficile*. NAAT detects sequences in the genes for toxin B, binary toxin and tcdC deletion nt 117 and can also detect the epidemic strain of *C. difficile* BI/NAP1/027.

Details of antibiotic use and other data were collected from the hospital information system.

### Statistical analysis.

Statistical analysis was done using IBM SPSS 20 (SPSS Inc, Chicago, USA). The results were given as percentage for all categorical variables. To determine a relationship between the 2 variables, Pearson’s coefficient and to compare the mean difference of numerical variables, independent sample t test were used.

## RESULTS

*Clostridium difficile* GDH assay was done on 660 stool samples. GDH enzyme was positive in 157 out of 660 (23.8%) samples. Moreover, *C. difficile* toxin was found to be positive in 58 out of 157 (36.9%) by ELFA. Total number of toxin positives was 58 out of 660 (8.8%) by ELFA. Six samples which were negative/ equivocal for toxin testing by ELFA were tested positive for *C. difficile* toxin by NAAT testing. The total number of toxin positives was 64 out of 660 (9.7%).

Among the 64 toxin positives, 42 (65.6%) were males and 22 (34.4%) females. Toxin positives were more commonly seen in the 61 to 80 years age group, accounting for 54.7% (35/64). Department wise distribution, antibiotic use, and risk factor distribution among the toxin positives are demonstrated in Tables 1, 2 and 3, respectively.

**Table 1.** Department wise distribution of toxin positives

**Department**	**Frequency (n = 64)**	**Percentage**
Gastroenterology	21	32.8
General medicine	18	28.1
Physical medicine	8	12.5
Oncology	5	7.8
Cardiology	4	6.3
Transplant	2	3.1
Nephrology	2	3.1
Endocrinology	2	3.1
Gynaecology	1	1.6
Pulmonary	1	1.6

**Table 2.** Antibiotic use among the 64 toxin positives

**Antibiotics**	**Frequency**	**Percentage**
Meropenem	35	54.7
Piperacillin-tazobactam	20	31.3
Colistin	18	28.1
Cefoperazone-sulbactam	15	23.4
Cefotaxime	14	21.9
Ciprofloxacin	13	20.3
Amikacin	13	20.3
Doxycycline	1	1.6

**Table 3.** Risk factor distribution among the 64 toxin positives

**Risk factor**	**Frequency**	**Percentage**
Hospitalization in past 60 days	64	100
PPI	39	60.9
Hypertension	34	53.1
Diabetes Mellitus	28	43.8
Malignancy	8	12.5
Chemotherapy	6	9.4

PPI-Proton Pump Inhibitors

Of the 64 toxin positive patients, 57 developed diarrhoea in the first 30 days after admission to the hospital, which accounted for 89.1%. Among the 93 GDH positive and toxin negative patients, 78 developed diarrhoea within 20 days of hospital admission, which accounted for 83.9% of these patients. There was a positive correlation between rise in GDH and toxin values among the 157 GDH positive samples (p value: 0.001). So, there was a statistically significant correlation between rise in GDH and toxin values. The mean GDH value was 8.45± 4.18 among toxin positives, and it was 4.23±4.26. (p value<0.001) among the toxin negatives, which indicated a statistically significant mean difference in GDH values between toxin positive and toxin negative patients. There was a statistically significant association among meropenem, colistin, cefotaxime, cefopera-zone-sulbactam, piperacillin-tazobactam, ciprofloxacin, amikacin and development of antibiotic associated diarrhea (p value <0.001).

In our study, there were 4 cases of colitis and 1 case of pseudomembranous colitis. Among the 64 toxin positives, 50 were on multiple antibiotics, which accounted for 78.1% of these patients. Fecal leucocytes were seen in 6.3% (4/64) of the *C. difficile* toxin positive patients. Of the toxin positives, 57 (89%) were treated with metronidazole, 4 (6.3%) with vancomycin, and the remaining 3 (4.7%) with a combination of vancomycin and metronidazole. Of the 64 toxin positives, 56 (87.5%) recovered, whereas 8 (12.5%) expired. Death was due to comorbidities like sepsis and malignancy. There was no significant difference in the toxin values among the expired patients, when compared to those who survived.

## DISCUSSION

*Clostridium difficile* is an established human pathogen that primarily causes gastroenteritis. Diagnosis of *C. difficile* associated diarrhoea (CDAD) is possible by history, clinical examination and detection of toxin in stool. In this study, CDAD was detected using *C. difficile* GDH (Glutamate dehydrogenase enzyme) assay, *C. difficile* toxin A and B assay, and Cepheid GeneXpert assay.

Prevalence of GDH antigen in other studies varied from 9.5% to 40.7%. Our finding of 23.8% was concordant with that of other studies. Brown et al. compared 4 different methods for diagnosing *C. difficile* infection and found that GDH antigen was present in 57 out of the 157 (16%) stool samples (8). GDH assay has a turnaround time of 1 hour and helps to rule out negative specimens and select specimens for further testing.

Prevalence of *C. difficile* toxin by ELFA was 8.8% and increased to an overall prevalence of 9.7%, with the inclusion of NAAT. Similar higher rates of *C. difficile* detection upon implementation of PCR based algorithms was reported by La Sala et al. (9). Prevalence of CDAD has been reported to be around 7% to 30% in patients with diarrhoea in different hospitals, based on several studies (10–13). The epidemic strain B1/NAP1/027 was not detected in any of the samples. In the current study, the toxin positive cases were mainly of male gender, advanced age group, and from gastroenterology and general medicine departments. Diabetes, hypertension, use of proton pump inhibitors, previous hospitalization, malignancy and chemotherapy were the risk factors seen in our study. Similar risk factors have been described in other studies. Segar et al. while conducting a study on the prevalence of *C. difficile* infection found that, 59% of the toxin positives were of male gender and 22% of the positive cases were from the general medicine department (14). Advanced age as a significant risk factor for developing severe CDAD was reported by Patel et al. (15). Eliakim-Raz et al. conducted a study on *C. difficile* infection in patients with diabetes and reported that 150 out of the total 486 (30.6%) patients were positive for *C. difficile* toxin (16). Gopal Rao et al. performed a study on CDAD among patients with lower respiratory tract infection and reported that the CDAD positive patients had a mean duration of hospital stay of 25.8 days (17). Niyogi et al. reported a prevalence of *C. difficile* toxin of 7% in children with acute diarrhoea (18). Our study found that only 3.1% (2/64) of the toxin positive patients belonged to the paediatric age group.

Analysis of antibiotic use among the positive cases in our study showed that most of them were on meropenem, cephalosporins, colistin, piperacillin-tazobactam, amikacin and ciprofloxacin prior to developing diarrhoea. Hensgens MP et al. performed a study on *C. difficile* infection after exposure to antibiotics and found that second and third generation cephalosporins and carbapenems were the strongest risk factors for developing CDI (19). Wieczorkiewicz et al. (20) reported fluoroquinolones as a risk factor for developing CDI. Studies around the world have shown varied results regarding the presence of fecal leucocytes in toxin positive stool samples. Barlett et al. while conducting a study on how to identify the cause of antibiotic associated diarrhoea, reported that fecal leucocyte test is useful in detecting CDAD (21). Savola et al. reported that fecal leucocyte test has no value in predicting *C. difficile* toxin positivity (22). We found fecal leucocytes in 6.3% of the toxin positive patients. Most of our positive cases were treated with metronidazole and the response rate was good. Mortality was low in our study. Zar et al. compared vancomycin and metronidazole in treating CDAD patients and found that treatment with either metronidazole or vancomycin resulted in clinical cure in mild CDAD cases, but vancomycin was superior in the case of severe CDAD (23).

This study had some limitations. Tissue culture cytotoxicity, which is the gold standard method in the diagnosis of *C. difficile* toxin, could not be done in this study and NAAT testing could only be done on 10 samples.

In conclusion, the total prevalence of *C. difficile* toxin in our hospital was 9.7%. Detection rates increased with inclusion of NAAT. Male gender, advanced age, diabetes, hypertension, proton pump inhibitors and previous hospitalization were risk factors for developing CDAD. There was a statistically significant association between the use of meropenem, ciprofloxacin, cefoperazone-sulbactam, cefotaxime, piperacillin-tazobactam, colistin, Amikacin and development of AAD. Generating local data is essential to create awareness of CDI among the physicians in a hospital. Quicker laboratory results of *C. difficile* can provide valuable clue to clinicians regarding antibiotic policies.
